# Relationship of Glycated Hemoglobin Levels with Myocardial Injury following Elective Percutaneous Coronary Intervention in Patients with Type 2 Diabetes Mellitus

**DOI:** 10.1371/journal.pone.0101719

**Published:** 2014-07-02

**Authors:** Xiao-Lin Li, Jian-Jun Li, Yuan-Lin Guo, Cheng-Gang Zhu, Rui-Xia Xu, Sha Li, Ping Qing, Na-Qiong Wu, Li-Xin Jiang, Bo Xu, Run-Lin Gao

**Affiliations:** Division of Dyslipidemia, State Key Laboratory of Cardiovascular Disease, Fu Wai Hospital, National Center for Cardiovascular Diseases, Chinese Academy of Medical Sciences and Peking Union Medical College, XiCheng District, Beijing, China; Virgen Macarena University Hospital, School of Medicine, University of Seville, Spain

## Abstract

**Background:**

Glycated hemoglobin (HbA1c) predicts clinical cardiovascular disease or cardiovascular mortality. However, the relationship between HbA1c and myocardial injury following elective percutaneous coronary intervention (PCI) in patients with type 2 diabetes mellitus (DM) has not been investigated.

**Objectives:**

The study sought to assess the relationship between HbA1c and myocardial injury following elective PCI in patients with type 2 DM.

**Methods:**

We studied a cohort of consecutive 994 diabetic patients with coronary artery disease (CAD) undergoing elective PCI. Periprocedural myocardial injury was evaluated by analysis of troponin I (cTnI). The association between preprocedural HbA1c levels and the peak values of cTnI within 24 hours after PCI was evaluated.

**Results:**

Peak postprocedural cTnI >1×upper limit of normal (ULN), >3×ULN and >5×ULN were detected in 543 (54.6%), 337 (33.9%) and 245 (24.6%) respectively. In the multivariate model, higher HbA1c levels were associated with less risk of postprocedural cTnI >1×ULN (odds ratio [OR], 0.85; 95% confidence interval [CI], 0.76–0.95; P = 0.005). There was a trend that higher HbA1c levels were associated with less risk of postprocedural cTnI >3×ULN (OR, 0.90; 95% CI, 0.81–1.02; P = 0.088). HbA1c was not associated with the risk of postprocedural cTnI elevation above 5×ULN (OR, 0.95; 95% CI, 0.84–1.08; P = 0.411).

**Conclusions:**

The present study provided the first line of evidence that higher preprocedural HbA1c levels were associated with less risk of myocardial injury following elective PCI in diabetic patients.

## Introduction

Glycated hemoglobin (HbA1c) is an index of metabolic control of diabetes, and reflects average blood glucose levels over the previous 2–3 months, including postprandial increases in the blood glucose level [1], [2]. There was compelling evidence suggested that the level of HbA1c predicted clinical cardiovascular disease or cardiovascular mortality [3]–[6]. However, the optimal glycemic control of diabetic patients with cardiovascular diseases was not well characterized. ADA, coupled with AHA and ACC just recommend less stringent HbA1c goals for diabetic patients with advanced macrovascular complications [7]. With the introduction of drug-eluting stents, the proportion of diabetic patients with coronary artery disease (CAD) who received percutaneous coronary intervention (PCI) is increasing. However, PCI is frequently accompanied with cardiac marker elevation after procedure or known as myocardial injury or infarction related to PCI [8], [9]. To date, we are not aware of any studies elucidating the impact of preprocedural glycemic control on periprocedural myocardial injury or infarction in patients with type 2 DM who underwent elective PCI. Thus, the aim of this study was to characterize the relation between HbA1c and periprocedural myocardial injury or infarction in patients with type 2 DM undergoing elective PCI.

## Methods

### Study population

The study complied with the Declaration of Helsinki, and was approved by the hospital ethnic review board (Fu Wai Hospital & National Center for Cardiovascular Diseases, Beijing, China). Informed written consent was obtained from all patients included in this study.

Between December 2010 and December 2012, 1032 consecutive diabetic patients with normal levels of cardiac troponin I (cTnI) and creatine kinase-MB (CK-MB) and without acute myocardial infarction in the past 4 weeks who attempt to undergo elective PCI at our center were eligible for this study. Of these patients, 33 patients were excluded because a total or subtotal chronic occlusion could not be crossed with a wire, 2 patients were excluded because a severely calcified or tortuous lesion could not be crossed with a balloon, 3 patients were excluded because treated with atheroablative, distal protection devices or aspiration thrombectomy. None of the patients died in the hospital. Thus, 994 patients were effectively included in the present study.

Adult patients with type 2 diabetes were identified based on recorded type 2 diabetes diagnosis or a prescription for oral hypoglycemic medication or insulin. Angiographic success of PCI was defined as residual stenosis less than 20% with stenting and residual stenosis less than 50% with balloon angioplasty only by visual estimation. Unstable angina was defined as rest angina, new-onset severe angina and increasing angina within 2 months. Periprocedural myocardial injury was defined as postprocedural cTnI >1×ULN. Secondly, postprocedural cTnI >3×ULN which was the diagnosis criteria of periprocedural myocardial infarction published in 2007 and postprocedural cTnI >5×ULN which was a requirement in the arbitrarily revised diagnosis criteria published in 2012 were also examined in this study [10], [11].

### Percutaneous coronary intervention

The indication for PCI was based on the ACC/AHA recommendations and was performed by experienced interventional cardiologists. Before the procedure, all patients without contraindications received aspirin 100 mg daily or a loading dose of 300 mg depending on whether already taken daily aspirin therapy, and received clopidogrel 75 mg daily or a loading dose of 300 mg depending on whether already taken daily long-term clopidogrel therapy prior to intervention. All patients received either 5000 U or 70 U/kg bolus of unfractionated heparin just before procedure and an additional bolus of 2,000 to 3,000 U were given every hour if the procedure lasted for more than an hour. Vascular access and PCI type (angioplasty only, angioplasty and stenting, or primary stenting) were determined by the interventional cardiologist according to patients' characteristics. Total balloon inflation times and inflation pressures were determined by the interventional cardiologist according to the technical properties of the balloon and the stent. After the procedure, all patients continued with aspirin and clopidogrel therapy daily. Use of glycoprotein IIb/IIIa receptor antagonists or anticoagulants was at the discretion of the interventional cardiologist.

### Electrocardiogram monitoring

All patients received a 12-lead Electrocardiogram record before, immediately after PCI, and in the case of the occurrence of symptoms which were interpreted as postprocedural ischaemic events. All patients received continuous Electrocardiogram monitoring using wireless technology after PCI during hospitalization.

### Biochemical measurements

Fasting venous blood samples were obtained immediately before intervention for measurement of fasting glucose levels, HbA1c, CK-MB activity and lipid profile. Plasma HbA1C and glucose was determined with conventional standard techniques. Cardiac troponin I (cTnI) levels were determined in venous blood samples before PCI, 24 hours after PCI, and in the event of the occurrence of symptoms or signs suggestive of myocardial ischemia. cTnI was analyzed by an immunochemiluminometric assay (Access AccuTnI, Beckman Coulter, California). The upper limit of normal (ULN) was defined as the 99th percentile of normal population with a total imprecision of <10%. The ULN of this test was 0.04 ng/ml. The peak value of cTnI within 24 hours after procedure was used for statistical analysis.

### Statistics

The baseline characteristics are presented according to the quartiles of HbA1c. Data are presented as mean ± SD, median with interquartile ranges, or frequencies with percentages, as appropriate. Comparisons among the HbA1c quartiles were made with analysis of variance, chi-square test, Fisher's exact test, Kruskal–Wallis test or Friedman test as appropriate. Univariate linear regression analyses were performed to determine the relation between clinical parameters and postprocedural cTnI levels. Variables with a *P* value <0.2 in the univariate linear regression were entered into a stepwise multivariable linear model to determine the independent predictive value of clinical parameters for postprocedural cTnI levels. Successful normalization of cTnI after log-transformation was evaluated using Kolmogorov-Smirnov test.

Logistic regression analyses were performed to determine the relationship of HbA1c with the occurrence of postprocedural cTnI elevations above various multiples of ULN. Logistic models were adjusted for variables independently associated with postprocedural cTnI levels. HbA1c was examined in quartiles and as continuous variables. A 2-tailed *P* value of <0.05 was considered statistically significant. All analyses were performed using SPSS version19.0 software (Chicago,Illinois,USA).

## Results

### Baseline characteristics according to quartiles of HbA1c

Baseline clinical characteristics according to quartiles of HbA1c were shown in Table 1. Fasting glucose, low-density lipoprotein cholesterol, C-reactive protein and triglycerides increased across the quartiles of HbA1c. Current smoking was more frequent in subjects with high HbA1c. There were no significant differences in distribution of sex, body mass index, hypertension, dyslipidemia, family history of CAD, unstable angina, prior MI, prior PCI, prior coronary artery bypass graft, high-density lipoprotein cholesterol, NT-proBNP, hemoglobin, preprocedural cTnI and medications at study entry among quartiles of HbA1c. Fasting glucose was highly correlated with HbA1c (r = 0.543, *P*<0.001). Higher quartiles of HbA1c were associated with less incidence of fasting glucose below 5 mmol/L (21.7%, 14.3%, 6.6% and 6.5% respectively; P<0.001).

**Table 1:** Baseline clinical characteristics.

	HbA1c at baseline				
Variable	Quartile 1 (n = 258)	Quartile 2 (n = 245)	Quartile 3 (n = 259)	Quartile 4 (n = 232)	*P* value
HbA1c, %	6.17±0.34	6.74±0.12	7.38±0.29	9.13±1.01	<0.001
Glucose, mmol/L	5.74±1.16	6.09±1.08	6.83±1.42	8.76±2.95	<0.001
Age, years	59.39±9.49	60.84±9.34	59.56±8.65	58.49±8.31	0.039
Male, n (%)	186 (72.1)	166 (67.8)	181 (69.9)	163 (70.3)	0.769
BMI, kg/m2	26.15±3.09	26.65±2.95	26.79±3.32	26.76±3.30	0.080
Hypertension, n (%)	193 (74.8)	175 (71.4)	187 (72.2)	157 (67.7)	0.374
Dyslipidemia, n (%)	227 (88.0)	211 (86.1)	218 (84.2)	198 (85.3)	0.651
Current smoker, n (%)	59 (22.9)	79 (32.2)	78 (30.1)	79 (34.1)	0.034
FH, n (%)	63 (24.4)	57 (23.3)	62 (23.9)	40 (17.2)	0.202
UA, n (%)	136 (52.7)	144 (58.8)	136 (52.5)	125 (53.9)	0.460
Prior MI, n (%)	61 (23.6)	60 (24.5)	57 (22.0)	57 (24.6)	0.898
Prior PCI, n (%)	80 (31.0)	69 (28.2)	76 (29.3)	72 (31.0)	0.877
Prior CABG, n (%)	8 (3.1)	9 (3.7)	7 (2.7)	6 (2.6)	0.896
LDL-C, mg/dl	89.78±31.61	92.92±29.47	94.85±31.18	100.12±33.83	0.003
HDL-C, mg/dl	40.36±9.77	40.74±9.18	41.42±11.97	39.59±8.61	0.234
Triglyceride, mg/dl	132.0 (92.8–177.1)	140.8 (107.2–178.5)	138.2 (98.3–199.3)	143.0 (109.8–207.9)	0.024
hs-CRP, mg/L	1.42 (0.83–2.66)	1.76 (1.07–3.92)	1.76 (0.97–2.96)	2.26 (1.14–4.09)	<0.001
NT-proBNP, fmol/ml	515.1 (410.0–683.3)	533.9 (424.5–699.7)	513.9 (413.5–683.7)	564.8 (461.3–732.7)	0.053
Hemoglobin, g/L	138.96±15.01	138.25±14.65	138.81±14.56	140.71±14.47	0.295
cTnI, ng/ml	0.005 (0.002–0.009)	0.004 (0.002–0.009)	0.005 (0.002–0.009)	0.006 (0.002–0.010)	0.097
Medications					
Statins, n (%)	256 (99.2)	242 (98.8)	254 (98.1)	228 (98.3)	0.687
Aspirin, n (%)	256 (99.2)	243 (99.2)	259 (100.0)	232 (100.0)	0.270
Clopidogrel, n (%)	258 (100.0)	245 (100.0)	259 (100.0)	232 (100.0)	-
β-Blockers, n (%)	229 (88.8)	218 (89.0)	224 (86.5)	209 (90.1)	0.641
Nitrates, n (%)	244 (94.6)	240 (98.0)	249 (96.1)	224 (96.6)	0.252
CCBs, n (%)	138 (53.5)	135 (55.1)	133 (51.4)	114 (49.1)	0.585
ACEIs, n (%)	79 (30.6)	78 (31.8)	82 (31.7)	75 (32.3)	0.981
ARBs, n (%)	87 (33.7)	81 (33.1)	88 (34.0)	85 (36.6)	0.854
Trimetazidine, n (%)	60 (23.38)	67 (27.3)	76 (29.3)	56 (24.1)	0.368

Values are expressed as mean ± SD, median with interquartile range or n (%).

LDL-C  =  low-density lipoprotein cholesterol; MI  =  myocardial infarcton; PCI  =  percutaneous coronary intervention; CABG  =  coronary artery bypass graft; CAD  =  coronary artery disease; HDL-C  =  high-density lipoprotein cholesterol; hs-CRP  =  high-sensitivity C-reactive protein; NT-proBNP  =  N-terminal pro-brain natriuretic peptide; cTnI  =  cardiac troponin I; CCBs  =  calcium channel blockers; ACE  =  angiotensin-converting enzyme; ARBs  =  angiotensin receptor blockers.

Procedural characteristics according to quartiles of HbA1c were shown in Table 2. Patients with higher HbA1c levels were more likely to receive more postdilatation. There were no significant differences in vascular access, target vessel, target lesion site and target lesion type among quartiles of HbA1c. There were also no significant differences in number of stents, total stent length, predilation times, maximum inflation pressure and maximum inflation time among quartiles of HbA1c.

**Table 2:** Procedural characteristics.

	HbA1c at baseline				
Variable	Quartile 1 (n = 258)	Quartile 2 (n = 245)	Quartile 3 (n = 259)	Quartile 4 (n = 232)	*P* value
Transradial access, n (%)	234 (90.7)	220 (89.8)	237 (91.5)	216 (93.1)	0.622
Target vessel					0.748
LM	9	16	13	7	
LAD	138	141	153	128	
LCX	90	75	79	76	
RCA	98	103	99	98	
Grafts	0	2	1	1	
Lesion location					0.268
Proximal	130	147	151	151	
Middle	195	175	201	173	
Distal	82	79	69	75	
branch	78	56	82	58	
Lesion classification					0.365
ACC/AHA type A/B1	84	73	66	69	
ACC/AHA type B2/C	290	288	315	266	
Bifurcation lesions, n (%)	108 (41.9)	97 (39.6)	117 (45.2)	97 (41.8)	0.648
Use with kissing balloon, n (%)	14 (5.4)	22 (9.0)	23 (8.9)	20 (8.6)	0.384
Occlusion lesions, n (%)	32 (12.4)	36 (14.7)	51 (19.7)	28 (12.1)	0.058
In-stent restenosis, n (%)	16 (6.2)	11 (4.5)	14 (5.4)	10 (4.3)	0.759
Number of stents implanted	1.88±1.01	2.05±1.05	2.04±1.07	2.03±1.03	0.178
Total stent length, mm	40.89±27.20	45.99±24.97	46.88±29.08	46.00±27.09	0.051
Maximum pressure, atm	17.60±3.69	18.26±3.89	17.76±3.46	17.95±3.66	0.215
Maximum inflation time, s	10.58±4.04	10.29±4.74	10.13±3.65	10.03±3.51	0.443
Number of predilation	2 (1–4)	2 (1–5)	3 (1–6)	3 (2–5)	0.054
Number of postdilatation	3 (2–6)	4 (2–6)	4 (2–6)	4 (2–7)	0.042
Postprocedural medication					
LMWH, n (%)	170 (65.9)	167 (68.2)	176 (68.0)	159 (68.5)	0.922
GPI, n (%)	44 (17.1)	44 (18.0)	31 (12.0)	34 (14.7)	0.239

Values are expressed as n (%), mean ± SD or median with interquartile range.

HbA1c  = glycated hemoglobin; LM  =  left main; LAD  =  left anterior descending; LCX  =  left circumflex; RCA  =  right coronary artery; LMWH  =  low molecular weight heparin; GPI  =  glycoprotein inhibitors.

### Association between HbA1c levels and postprocedural cTnI levels

There was a similar trend that lower preprocedural HbA1c and fasting glucose levels were associated with higher postprocedural cTnI levels in the simple regression analysis (Table 3). Simple regression analyses revealed that age, prior MI, NT-proBNP, creatinine, preprocedural cTnI, number of target vessels, number of B2/C type lesions, number of bifurcation lesions, number of predilation, number of postdilation, use of kissing balloon, maximum inflation pressure, number of stents and total stent length were positively associated with postprocedural cTnI levels, whereas high hemoglobin levels were associated with low postprocedural cTnI levels.

**Table 3:** Analysis of factors related to postprocedural cTnI levels (log-transformed).

	Simple Regression		Multiple Regression	
Variable	Standard coefficient	*P* value	Standard coefficient	*P* value
Age	0.125	<0.001	0.117	<0.001
Male	-0.023	0.473		
Unstable angina	-0.058	0.065		
Prior MI	0.095	0.003	0.076	0.012
Prior PCI	-0.015	0.640		
Prior CABG	0.008	0.791		
Hypertension	0.030	0.346		
Current smoking	0.011	0.739		
Family history of CAD	0.021	0.517		
HbA1C	-0.053	0.097	-0.071	0.018
Glucose	-0.053	0.095		
LDL-C	0.043	0.177		
HDL-C	0.005	0.866		
Triglyceride	0.007	0.833		
hs-CRP	-0.014	0.664		
NT-proBNP	0.117	<0.001	0.080	0.009
Creatinine	0.069	0.030		
Hemoglobin	-0.064	0.043		
Preprocedural cTnI	0.124	<0.001	0.086	0.005
Number of target vessels	0.220	<0.001	0.131	<0.001
Number of B2/C type lesions	0.201	<0.001		
Number of bifurcation lesions	0.132	<0.001		
Number of occlusion lesions	0.013	0.680		
Number of in-stent restenosis	-0.022	0.485		
Number of predilation	0.182	<0.001		
Number of postdilatation	0.201	<0.001	0.075	0.043
Use of kissing balloon	0.089	0.005		
Maximum inflation pressure	0.075	0.018		
Maximum inflation time	0.045	0.157		
Number of stents	0.248	<0.001		
Total stent length	0.252	<0.001	0.152	<0.001

MI  =  myocardial infarcton; PCI  =  percutaneous coronary intervention; CABG  =  coronary artery bypass graft; CAD  =  coronary artery disease; LDL-C  =  low-density lipoprotein cholesterol; HDL-C  =  high-density lipoprotein cholesterol; hs-CRP  =  high-sensitivity C-reactive protein; NT-proBNP  =  N-terminal pro-brain natriuretic peptide; cTnI, cardiac troponin I.

Stepwise multivariable analysis revealed that factors independently associated with postprocedural cTnI levels were age, prior myocardial infarction, NT-proBNP, preprocedural cTnI, number of target vessels, number of postdilation and total stent length were positively associated with postprocedural cTnI levels, whereas HbA1c levels were inversely associated with postprocedural cTnI levels (Table 3).

### Association between HbA1c levels and postprocedural cTnI elevation

**Figure 1:**
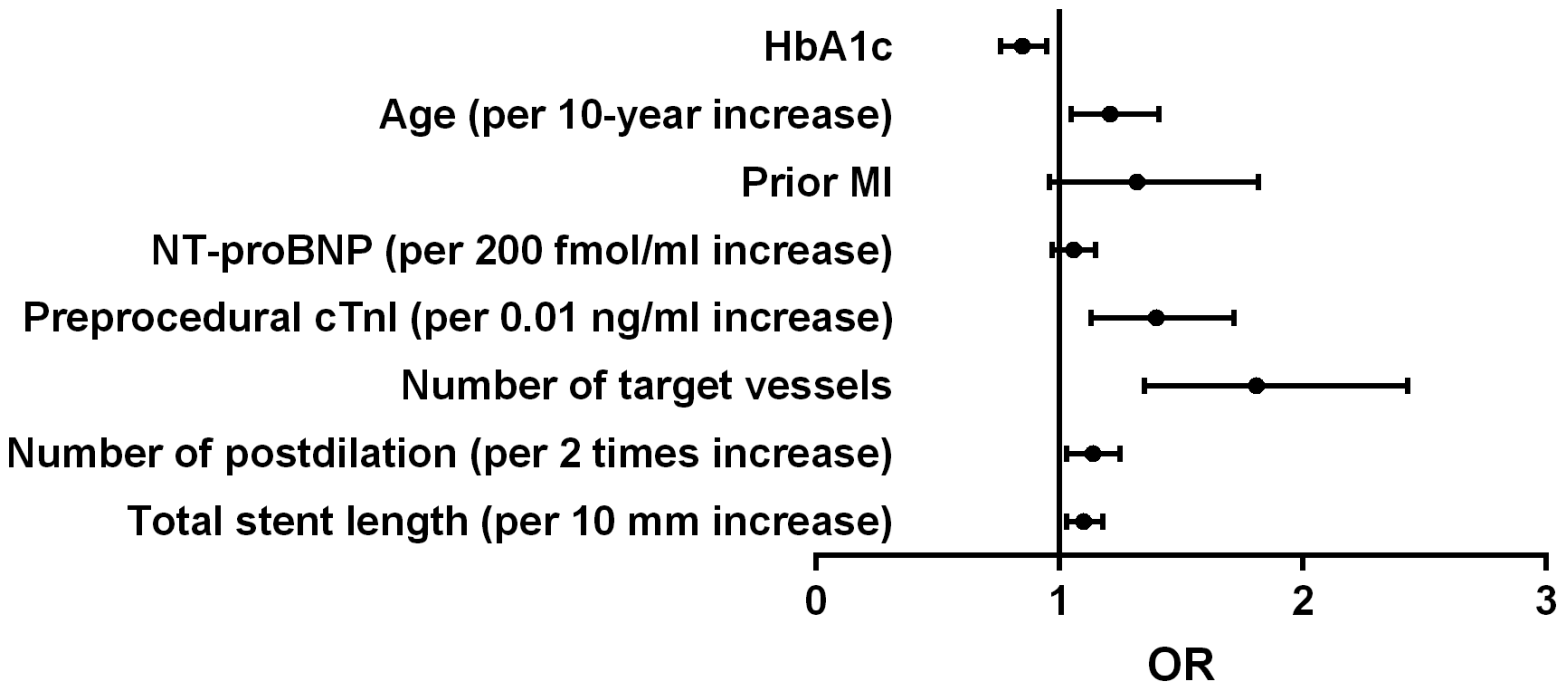
Odds ratio for postprocedural cTnI elevation above 1×ULN. HbA1c  = glycated hemoglobin; cTnI  =  cardiac troponin I; OR  =  odds ratio; ULN  =  upper limit of normal; MI  =  myocardial infarcton; NT-proBNP  =  N-terminal pro-brain natriuretic peptide.

**Figure 2:**
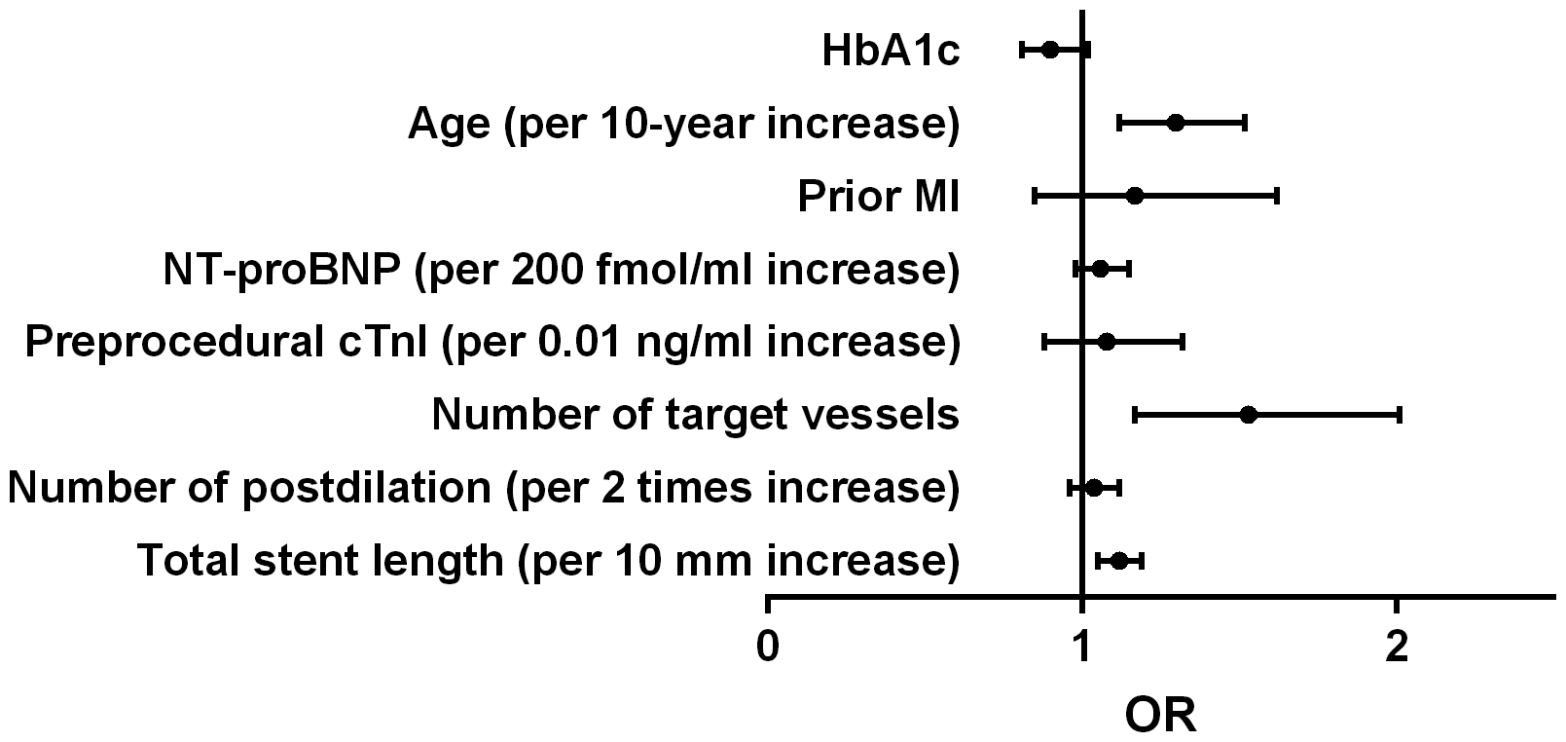
Odds ratio for postprocedural cTnI elevation above 3×ULN. HbA1c  = glycated hemoglobin; cTnI  =  cardiac troponin I; OR  =  odds ratio; ULN  =  upper limit of normal; MI  =  myocardial infarcton; NT-proBNP  =  N-terminal pro-brain natriuretic peptide.

**Figure 3:**
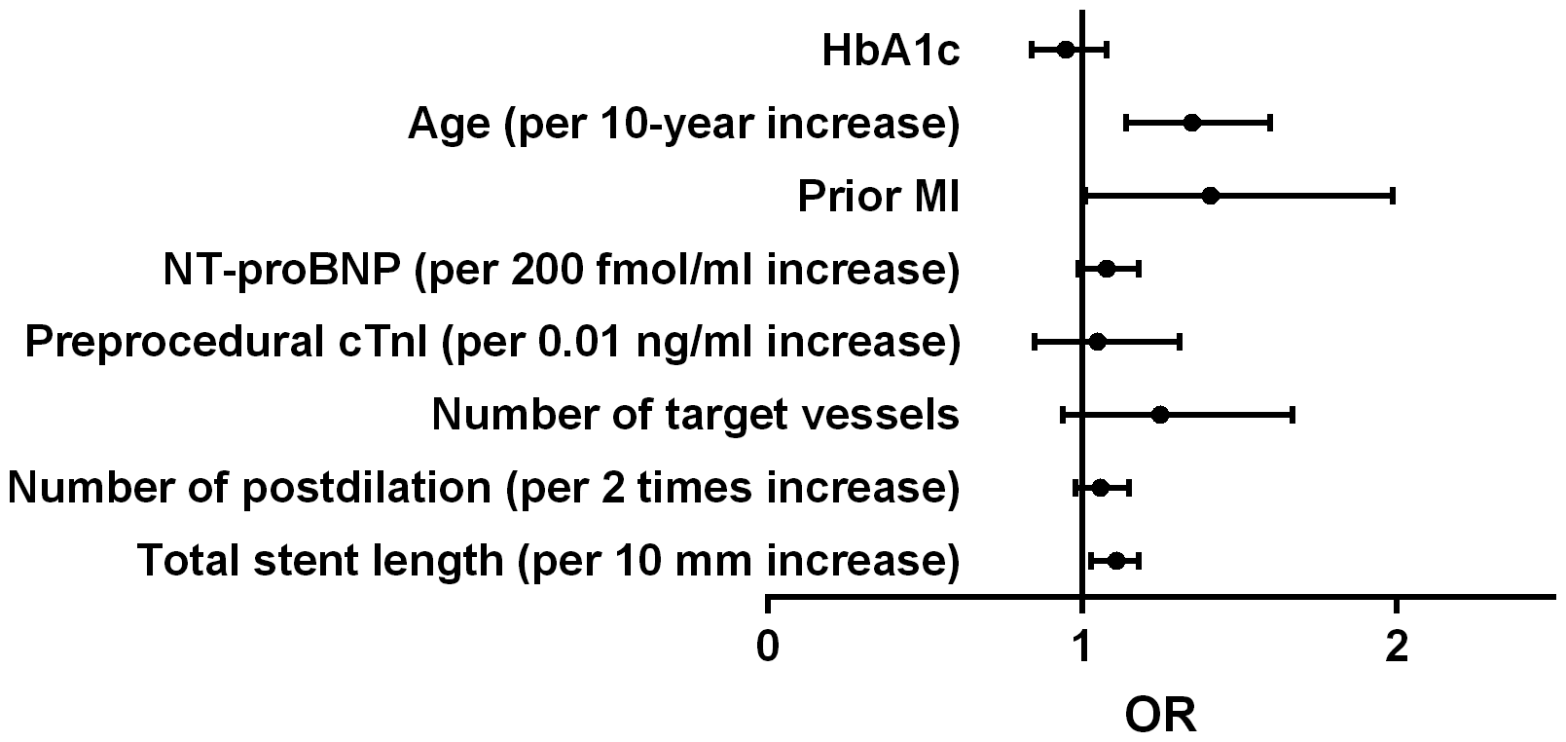
Odds ratio for postprocedural cTnI elevation above 5×ULN. HbA1c  = glycated hemoglobin; cTnI  =  cardiac troponin I; OR  =  odds ratio; ULN  =  upper limit of normal; MI  =  myocardial infarcton; NT-proBNP  =  N-terminal pro-brain natriuretic peptide.

Peak postprocedural cTnI >1×ULN, >3×ULN and >5×ULN were detected in 543 (54.6%), 337 (33.9%) and 245 (24.6%) respectively. Calculating HbA1c as a continuous variable, simple logistic regression showed that each increment of 1% in the HbA1c level was associated with less risk of postprocedural cTnI elevation above 1×ULN (Table 4). HbA1c was not significantly associated with postprocedural cTnI elevation above 3×ULN and 5×ULN. After adjusting for covariates, each increment of 1% in the HbA1c level was more strongly associated with less risk of postprocedural cTnI elevation above 1×ULN (Table 4, Figure 1). There was a trend that each increment of 1% in the HbA1c level was associated with less risk of postprocedural cTnI elevation above 3×ULN, but this did not reach a statistical significance (Table 4, Figure 2). HbA1c was not significantly associated with postprocedural cTnI elevation above 5×ULN (Table 4, Figure 3).

**Table 4:** Odds ratio for postprocedural cTnI elevation associated with 1% increment in the HbA1c.

	cTnI elevation >1×ULN		cTnI elevation >3×ULN		cTnI elevation >5×ULN	
Variable	OR (95%CI)	*P*	OR (95%CI)	*P*	OR (95%CI)	*P*
**Unadjusted model**						
HbA1C	0.90(0.81–0.99)	0.034	0.92(0.83–1.03)	0.151	0.96(0.85–1.09)	0.547
**Adjusted model**						
HbA1C	0.85(0.76–0.95)	0.004	0.90(0.81–1.02)	0.088	0.95(0.84–1.08)	0.411
Age	1.02(1.01–1.04)	0.011	1.03(1.01–1.04)	0.001	1.03(1.01–1.05)	0.001
Prior MI	1.32(0.96–1.82)	0.088	1.17(0.85–1.62)	0.344	1.41(1.01–1.99)	0.049
NT-proBNP	1.00(1.00–1.01)	0.192	1.00(1.00–1.01)	0.162	1.00(1.00–1.01)	0.075
Preprocedural cTnI	1.03(1.01–1.06)	0.002	1.01(0.99–1.03)	0.456	1.01(0.98–1.03)	0.632
Number of target vessels	1.81(1.35–2.43)	<0.001	1.53(1.17–2.01)	0.002	1.25(0.94–1.67)	0.128
Number of postdilatation	1.07(1.02–1.12)	0.008	1.02(0.98–1.06)	0.383	1.03(0.99–1.07)	0.180
Total stent length	1.01(1.00–1.02)	0.005	1.01(1.00–1.02)	0.001	1.01(1.00–1.02)	0.004

HbA1c  = glycated hemoglobin; cTnI  =  cardiac troponin I; OR  =  odds ratio; ULN  =  upper limit of normal; MI  =  myocardial infarcton; NT-proBNP  =  N-terminal pro-brain natriuretic peptide.

### Association between HbA1c quartiles and postprocedural cTnI levels

The interquartile ranges of postprocedural cTnI levels for each quartile of HbA1c were 0.052 (0.018–0.203), 0.063 (0.021–0.184), 0.049 (0.015–0.217) and 0.041 (0.012–0.198) respectively. There is a linear trend that higher quartiles of HbA1c were associated with lower postprocedural cTnI levels. After multivariate adjustment for other factors which were independently associated with postprocedural cTnI levels, higher quartiles of HbA1c were significantly associated with lower postprocedural cTnI levels (Table 5). And higher quartiles of HbA1c were associated with less risk of postprocedural cTnI elevation above 1×ULN. There was a trend that higher quartiles of HbA1c were associated with less risk of postprocedural cTnI elevation above 3×ULN, but this did not reach a statistical significance. The quartiles of HbA1c were not associated with the risk of postprocedural cTnI elevation above 5×ULN (Table 6).

**Table 5:** Distribution of post-PCI cardiac troponin I.

	HbA1c at baseline					
Variable	Quartile 1 (n = 258)	Quartile 2 (n = 245)	Quartile 3 (n = 259)	Quartile 4 (n = 232)	β	*P* for trend
Post-PCI cTnI, ng/ml	0.052(0.018–0.203)	0.063(0.021–0.184)	0.049(0.015–0.217)	0.041 (0.012–0.198)	−0.073	0.014
Post-PCI cTnI elevation						
>1×ULN, n (%)	147 (57.0)	140 (57.1)	139 (53.7)	117 (50.4)	−0.145	0.018
>3×ULN, n (%)	91 (35.3)	87 (35.5)	86 (33.2)	73 (31.5)	−0.088	0.166
>5×ULN, n (%)	66 (25.6)	56 (22.9)	65 (25.1)	58 (25.0)	−0.019	0.785

Values were expressed as median with interquartile ranges or n (%). The analyses were adjusted for age, prior myocardial infarction, NT-proBNP, preprocedural cTnI, number of target vessels and total stent length.

**Table 6:** Unadjusted and adjusted OR for periprocedural myocardial injury according to quartiles of HbA1c.

	cTnI elevation >1×ULN		cTnI elevation >3×ULN		cTnI elevation >5×ULN	
Variable	OR (95%CI)	*P*	OR (95%CI)	*P*	OR (95%CI)	*P*
**Unadjusted model**						
Quartile 1 (reference)						
Quartile 2	1.01(0.71–1.43)	0.970	1.01(0.70–1.46)	0.955	0.86(0.57–1.30)	0.476
Quartile 3	0.88(0.62–1.24)	0.449	0.91(0.63–1.31)	0.621	0.98(0.66–1.45)	0.899
Quartile 4	0.77(0.54–1.10)	0.147	0.84(0.58–1.23)	0.373	0.97(0.65–1.46)	0.883
**Adjusted model**						
Quartile 1 (reference)						
Quartile 2	0.86(0.59–1.25)	0.437	0.88(0.60–1.28)	0.490	0.74(0.49–1.13)	0.167
Quartile 3	0.74(0.52–1.08)	0.116	0.82(0.56–1.19)	0.293	0.88(0.59–1.33)	0.549
Quartile 4	0.65(0.44–0.95)	0.025	0.77(0.52–1.14)	0.184	0.90(0.59–1.37)	0.610
Age	1.02(1.01–1.04)	0.009	1.03(1.01–1.04)	0.001	1.03(1.01–1.05)	<0.001
Prior MI	1.31(0.95–1.80)	0.099	1.16(0.84–1.61)	0.362	1.42(1.01–2.00)	0.047
NT-proBNP	1.00(1.00–1.01)	0.201	1.00(1.00–1.01)	0.170	1.00(1.00–1.01)	0.082
Preprocedural cTnI	1.03(1.01–1.06)	0.002	1.01(0.99–1.03)	0.471	1.01(0.98–1.03)	0.682
Number of target vessels	1.79(1.34–2.41)	<0.001	1.53(1.17–2.00)	0.002	1.26(0.95–1.68)	0.114
Number of postdilatation	1.06(1.02–1.12)	0.009	1.02(0.98–1.06)	0.381	1.03(0.99–1.07)	0.183
Total stent length	1.01(1.00–1.02)	0.004	1.01(1.00–1.02)	0.001	1.01(1.00–1.02)	0.004

HbA1c  = glycated hemoglobin; cTnI  =  cardiac troponin I; OR  =  odds ratio; ULN  =  upper limit of normal; MI  =  myocardial infarcton; NT-proBNP  =  N-terminal pro-brain natriuretic peptide.

## Discussion

The present study provided the first line of evidence that higher preprocedural HbA1c levels were associated with less risk of myocardial injury following elective PCI in diabetic patients. Thus, our study provided the novel finding regarding the relationship between preprocedural HbA1c and periprocedural myocardial injury.

PCI has become an important strategy for patients with CAD. Patients with type 2 diabetes mellitus (DM) have a higher prevalence of CAD than the general population. Because of poor outcome, PCI in diabetic patients have been recognized as a complex procedure. With advances in PCI techniques and medications, especially with introduction of drug-eluting stents, more and more diabetic patients receive PCI. However, PCI was still frequently companied with postprocedural cardiac marker elevation. There was a large body of data correlating troponin elevation after elective PCI with adverse clinical outcomes [12]–[15]. Third universal definition of myocardial infarction has raised the diagnostic threshold of PCI-related myocardial infarction from the elevation of troponin above 3 times ULN to the elevation of troponin above 5 times ULN, and suggested that this threshold was arbitrarily chosen, based on clinical judgement and societal implications of the label of PCI-related myocardial infarction. Myocardial injury is used for postprocedural cTn value is >1×ULN and ≤5×ULN [16]. So, we used many different cTnI cut points. Although a number of studies have investigated the risk factors associated with periprocedural myocardial infarction or injury [9], [17], [18], less of them focused on diabetic patients or the impact of glycemic control on periprocedural myocardial infarction or injury in diabetic patients. And elevated HbA1c or poor glycemic control is associated with increased risk of cardiovascular events in diabetic patients [4]–[6], but whether elevated HbA1c is still associated with increased risk of myocardial infarction or injury following elective PCI in diabetic patients is still unknown.

In the present study, we included 994 diabetic patients undergoing elective PCI to determine the relation of preprocedural HbA1c levels with postprocedural cTnI elevation. Univariate analysis showed that some clinical and procedural characteristics were associated with postprocedural cTnI levels. There were almost identical pattern between the inverse associations of HbA1c and fasting glucose with postprocedural cTnI levels. However, after multivariate stepwise analysis, HbA1c was still in the model, but fasting glucose was not. HbA1c reflects both fasting and postprandial blood glucose levels over the previous 2–3 months, has less fluctuation individually than fasting blood glucose level, and can be measured in the nonfasting state. These characteristics may result that HbA1c outperform fasting glucose in prediction of periprocedural myocardial injury. Patients in the highest quartile of HbA1c were likely to receive more predilation and postdilation, and longer total stent length implanted. This maybe reflect high atherosclerotic burden in these patients with high HbA1c levels [19]. However, these patients experienced reduced risk of periprocedural myocardial injury. Regardless of calculating as quartiles or continuous variables, higher HbA1c levels were associated with less risk periprocedural myocardial injury. A U-shaped or J-shape association between HbA1c and periprocedural myocardial injury did not appear. Interestingly, there was also an inverse relationship between HbA1c levels and mortality in diabetic patients with advanced systolic heart failure [20].

The exact reasons for the relationship of lower HbA1c levels with periprocedural myocardial injury were unclear. There were some plausible explanations for this relationship. The energy supply of ischemic myocardium mainly dependents on anaerobic pathways and carbohydrate substrates [21]. Patients with lower HbA1c may have less energy supply for ischemic myocardium during or after procedure. The present study showed that HbA1c was highly correlated with fasting glucose. Libby and colleagues have showed that hypoglycemia increased myocardial damage during acute experimental coronary artery occlusion in dogs [22]. The study by Nusca et al showed that preprocedural glucose levels were inversely associated with periprocedural myocardial infarction in 572 patients of which 198 was diabetic [23]. The study by Madani et al showed that low preprocedural glucose levels were associated with increased incidence of periprocedural myocardial injury in 1012 patients undergoing elective PCI of which 260 was diabetic [24]. Unlike these studies, we examined the relation of fasting glucose and HbA1c with periprocedural myocardial injury in diabetic patients undergoing elective PCI, and found the superiority of HbA1c over fasting glucose for prediction of periprocedural myocardial injury. This was consistent with the study by Nicholls which showed a stronger correlation between plaque characteristics and glycated hemoglobin than fasting glucose [25]. Although high levels of HbA1c were associated with large plaque volume [19], higher HbA1c levels were still associated with less risk of periprocedural myocardial injury in our study. The positive association between HbA1c and glucose might partly explain the inverse relationship between HbA1c and periprocedural myocardial injury. The energy supply of ischemic myocardium mainly dependents on anaerobic pathways with carbohydrate substrates. Patients with hypoglycemia may be under greater energy stress and have less energy supply.

There were also some other reasons accounted for the inverse relationship between HbA1c and periprocedural myocardial injury in diabetic patients. Diabetic patients with high levels of HbA1c have increased coronary plaque calcification despite of large plaque volume [19], [26]. Calcified and hard plaques were difficult to be dilated and less likely to be crushed to debris during balloon dilation. The negative or unexpected results of several clinical trials which were designed to determine the effect of achieving a low HbA1c level on cardiovascular events, could also partly explain our results. Because of increased mortality in participants randomized to intensive glucose control to achieve an HbA1c level below 6%, Action to Control Cardiovascular Risk in Diabetes (ACCORD) was terminated early [27]. Both Action in Diabetes and Vascular Disease–Preterax and Diamicron Modified Release Controlled Evaluation (ADVANCE) and the Veterans Affairs Diabetes Trial (VADT) showed no significant reduction in cardiovascular events with intensive glucose control by which a mean HbA1c level of 6.3% and 6.9% were achieved respectively [28], [29]. To date, the optimal HbA1C target in diabetic patients with coronary artery disease is still a subject of ongoing controversy.

Despite the detailed data collection in this study, we acknowledged several potential limitations of our study. First, although we attempted to adjust for potential confounders, we can not exclude the possibility that unmeasured variables may have confounded results. Second, it has been suggested that CK-MB might have a better predictive value than troponins [30], but we did not measure the CK-MB levels after procedure due to insurance cost. However, troponins are more sensitive and specific biomarkers for myocardium than CK-MB activity and CK-MB mass, and the third universal definition of myocardial infarction has recommended troponin using for diagnosis of PCI-related myocardial infarction and injury. And there was also a large body of data demonstrated that postprocedural troponin elevation was associated with a worse clinical outcome [12]–[15]. Third, we conjectured that hypoglycemia might be one cause of periprocedural myocardial injury for patients with low HbA1c, but hypoglycemic episodes were not monitored in the current study. Fourth, the lack of a control (non-diabetic) group was also a limitation. Finally, a single center study may also be a limitation in our study. However, the present study has revealed a previously unrecognized relationship between HbA1c level and myocardial injury following elective PCI in diabetic patients.

## Conclusions

In summary, our prospective study in a large cohort of consecutive diabetic patient with CAD, for the first time, demonstrated that higher preprocedural HbA1c concentrations were linked with less risk of myocardial injury following elective PCI in patients with diabetes mellitus. Further prospective studies are needed to identify whether less stringent HbA1c goals before procedure is appropriate for diabetic patients undergoing elective PCI.
